# Reimagining drug regulation in the age of AI: a framework for the AI-enabled Ecosystem for Therapeutics

**DOI:** 10.3389/fmed.2025.1679611

**Published:** 2025-10-16

**Authors:** Rominder Singh, Karen Zhou, Jared R. Auclair

**Affiliations:** ^1^Northeastern University, Boston, MA, United States; ^2^Northeastern University, Toronto Campus, Toronto, ON, Canada

**Keywords:** artificial intelligence, drug regulations, regulatory harmonization, drug development, policy, human therapeutics, medicines

## Abstract

Artificial intelligence (AI) is increasingly integrated into drug development and regulatory decision-making; however, the regulatory landscape governing these technologies remains fragmented. While agencies such as the US Food and Drug Administration (FDA) and European Medicines Agency (EMA) have begun issuing guidance on AI applications in human therapeutics, these frameworks differ substantially in scope, terminology, and application. This lack of alignment complicates regulatory interpretation, creates barriers to regulatory coordination, and impedes equitable access to AI-enabled therapies. In this article, we introduce the AI-enabled Ecosystem for Therapeutics (AI2ET) framework, a conceptual and policy-oriented model designed to support the federation of regulatory knowledge and promote regulatory alignment. The AI2ET shifts regulatory focus from individual AI-generated products to the broader AI-enabled systems, platforms, and processes that underpin drug development. This approach addresses current regulatory gaps in AI oversight by articulating clear definitions of the components that constitute the ecosystem, establishing risk-based decision-making pathways, and finally offering regulatory guidance to navigate the ecosystem. The article offers six key policy recommendations that include strengthening international cooperation, establishing shared regulatory definitions, and investing in regulatory capacity building. By laying down a conceptual foundation for regulatory science-based oversight of AI in therapeutic development, the AI2ET framework offers a path forward for inclusive, effective, and equitable oversight of AI in regulating human therapeutics.

## Introduction

The integration of artificial intelligence (AI) in drug development is transforming the pharmaceutical industry. However, the current regulatory frameworks have yet to keep pace with the adoption of AI throughout the drug development cycle. While traditional drug development, which involves extensive preclinical studies, multi-phase clinical trials, and regulatory review, is known for its lengthy and resource-intensive process, AI is challenging this conventional paradigm by accelerating discovery, streamlining drug development, and reducing costs through predictive modeling, automation, and real-time data analysis (1). Although definitions of AI can vary, it is commonly understood as the development of computer systems capable of performing tasks that typically require human intelligence, such as learning, reasoning, problem-solving, and decision-making (2). Predictive AI has long played a role in drug development. More recently, generative AI has emerged as a transformative force, particularly in healthcare and medicine, by generating new content and insights from large datasets (3).

Regulatory frameworks are emerging in response to these changes (4). For example, regulatory guidance has been issued in the United States (US) and European Union (EU) outlining a risk-based framework for evaluating AI models used in drug and biologic development, focusing on transparency, data quality, and human oversight. These efforts reflect a growing recognition of the need to balance innovation with patient safety (5, 6). Yet, the existing regulatory framework remains inadequate in addressing the broad, adaptive nature of AI systems in drug development.

As AI becomes increasingly embedded across the therapeutic lifecycle, from generative models designing novel drug candidates to adaptive systems optimizing manufacturing processes, there is a growing need for structured, forward-looking regulatory frameworks.

This article proposes the concept of the *AI-Enabled Ecosystem for Therapeutics (AI2ET)* as a potential solution to regulatory fragmentation. Regulatory agencies must evolve beyond the current fragmented approach rooted in a conventional paradigm that treats AI as discrete tools by adopting an integrated framework that recognizes the ecosystem created using AI in drug development.

## The core challenge: regulatory framework fragmentation

### Current state of regulation

The current AI regulatory landscape is inconsistent, creating regulatory fragmentation. For example, while the US Food and Drug Administration (FDA) has been developing guidelines for AI-enabled medical devices since at least 2019, such as guidelines on good machine learning practices, algorithmic transparency, and predetermined change control. And yet, the agency issued its first major guidance specific to AI in drug and biologics development in January 2025 (6). Traditional drug development follows well-established regulatory pathways developed over decades of precedent. However, AI integration in therapeutics presents unforeseen challenges to the established pathways.

Moreover, the FDA currently applies differing regulatory frameworks to artificial intelligence depending on the application context. For AI-enabled medical devices, the AI itself is subject to direct evaluation (7). This includes assessments of algorithm transparency, performance, data integrity, and lifecycle management, particularly for adaptive models, which may require ongoing oversight under a predetermined change control plan (8). By contrast, in the context of AI-generated therapeutics, oversight is centered on the safety, efficacy, and clinical validation of the final drug product, with AI treated as a component of the development process rather than a regulated entity (9). AI tools used in drug development face fragmented oversight from discovery through manufacturing under various existing frameworks, including Good Clinical Practice (GCP) and Good Manufacturing Practice (cGMP). As a result, the differential regulation of AI for medical devices and drugs in the current regulatory landscape creates fragmentation.

AI-related regulatory fragmentation also refers to the increasing inconsistency in how artificial intelligence is regulated globally. Over 70 countries have introduced national AI policies, but these frameworks often differ in scope, terminology, and application to drug regulations (10). Fragmentation is also evident within countries, where medical devices, biotherapeutics, and digital health fall under separate regulatory pathways, leading to disjointed oversight of AI-enabled systems (4, 8, 9). Additionally, while some agencies like the FDA and European Medicines Agency (EMA) have advanced draft guidance, many regions, especially low- and middle-income countries, lack AI-related drug and device regulation (11).

In July 2025, the US issued the White House AI Action Plan, outlining over 90 federal actions aimed at boosting AI innovation, building national infrastructure, and establishing US leadership in global AI standards (12). For the biopharma industry, this may mark a significant shift. The plan promotes regulatory sandboxes and risk-based frameworks, with the FDA highlighted as a central player. This could ease regulatory barriers and accelerate the adoption of AI across drug development, clinical trials, and manufacturing. As the US takes the lead in setting global AI standards, biopharma companies may benefit from more flexible and harmonized regulatory pathways. As part of this effort, there’s an opportunity to establish broad, shared terminology like AI2ET, which could help unify how AI-enabled ecosystems are understood and regulated across the industry. However, the question remains if the rest of the world is harmonized with the US AI Action Plan.

### Why the need to address regulatory fragmentation

In the context of regulating AI for drug development, regulatory fragmentation creates several issues that pose barriers to achieving a coherent regulatory framework.

First, because of regulatory fragmentation, there is no consistent definition of AI. Although the FDA defines AI broadly as “a machine-based system that can, for a given set of human-defined objectives, make predictions, recommendations, or decisions influencing real or virtual environments.,” this definition spans all AI applications. This high-level definition has resulted in different levels of oversight based on existing frameworks instead of the technology’s characteristics or risk profile (13).

A second issue is the limitations of the Context of Use (CoU) framework. The FDA has recently introduced the CoU framework (6). The CoU concept was initially used for biomarkers (14) and is a foundational regulatory concept used by the FDA to define the specific circumstances under which a drug development tool or AI application is intended to be used. It outlines the tool’s purpose, scope, target population, and decision-making role, and serves as the basis for determining the appropriate level of regulatory oversight. By clearly articulating how and where a tool will be applied, the CoU helps ensure that validation efforts are aligned with real-world use and that regulatory evaluations remain risk-based and fit-for-purpose. However, how CoU is to be applied in regulatory decision making for AI-enabled drug development is unclear. Unanticipated products and systems are bound to be presented for review to the regulators. Since the regulators are known to regulate through precedent or what is already established, the novel AI-enabled approach could create a conundrum for the regulators where AI-generated therapeutics present novel mechanisms or outcomes that cannot be fully understood or explained (15). In drug applications that rely on unprecedented AI methodologies, regulators may choose to deny approval due to the absence of established frameworks or clear rationale. Alternatively, they may proceed under regulatory uncertainty, which can introduce delays and increase the burden of evidence, ultimately slowing access to therapies.

A third concern is that there is fragmentation in how AI-related drug regulations are being developed across the field of human therapeutics. For instance, recent FDA draft guidance (6) on the use of AI in regulatory decision-making for drugs and biologics excludes early-stage discovery and operational AI applications unless they directly impact patient safety. This guidance, while a step forward, highlights existing gaps in oversight and highlights the need for more comprehensive regulatory approaches across the AI-enabled ecosystem for human therapeutics.

Finally, there is a fragmentation in the terminology, guidelines, and application of AI-related drug regulations. Currently, most AI-related regulatory frameworks are being shaped by the US, EU and other high-income countries, often in isolation and with varying definitions and approaches. This lack of alignment creates barriers to future collaboration and consistent oversight across regions. Establishing a unified regulatory language and shared principles through international cooperation will be essential to promote transparency, enable equitable participation, and global availability of AI-enabled human therapeutics.

### Regulatory uncertainty

Regulatory fragmentation generates regulatory uncertainty for sponsors developing AI-enabled therapeutics. The use of AI in the drug development process challenges the traditional regulatory paradigm. The regulatory uncertainty applies to both the industry and regulators. There is a lack of guidance to evaluate AI-generated therapeutics that may rely on computational instead of experimental validation data or leverage algorithmic decision-making processes without transparency. Regulatory agencies will encounter unprecedented scenarios that do not fit neatly within the parameters of established review processes. The following examples illustrate the regulatory uncertainty in practice, prompting a need to adapt existing frameworks to accommodate the rapidly evolving AI-enabled drug development landscape.

### The case of Elsa going alone

In June 2025, the FDA launched Elsa, a generative AI assistant designed to support internal regulatory processes across its centers (16). This initiative represents a significant milestone in the agency’s broader strategy to integrate AI into the drug regulatory framework. Elsa is designed to assist with administrative and analytical tasks such as summarizing adverse event reports, reviewing clinical protocols, generating database code, and identifying inspection targets, all within a secure, cloud-based infrastructure. While the tool is not yet involved in regulatory decision-making, its deployment signals a growing institutional commitment to leveraging AI for greater efficiency and responsiveness in regulatory science. However, early implementation has revealed key challenges, including concerns around accuracy, consistency, and the potential for AI-generated content to “hallucinate” or misrepresent information. Elsa’s rollout is indicative of both the promise and complexity of integrating AI into regulatory ecosystems: it can streamline routine processes and reduce review timelines, but its role in substantive regulatory judgment remains constrained by the need for transparency, accountability, and trustworthiness. As such, Elsa exemplifies the cautious, yet deliberate steps regulators can take toward developing AI-enabled regulatory infrastructure. While pharmaceutical firms are closely monitoring Elsa’s deployment, as its use may shape future review standards and expectations, as of this writing, there is no direct collaboration or shared development between Elsa and external sponsor entities or other global regulatory bodies.

### AI-guided human therapeutics development: an example of a regulatory challenge

Consider a hypothetical, yet increasingly plausible scenario: an Investigational New Drug (IND) application is submitted to the FDA for a synthetic protein therapeutic that is fully designed using advanced AI models, drawing on innovations that were awarded the 2024 Nobel Prize in Medicine (17). These investigational human therapeutics may have no natural counterpart, and the target was validated using silico methods, bypassing animal toxicology studies in favor of AI-guided digital twin simulations. As the scientific capabilities to create entirely new classes of molecules advance, the regulatory frameworks tasked with evaluating them are being stretched beyond their original intent. In such cases, the mechanisms of action may be novel, the safety data modeled rather than observed, and the decision-making pathways of the AI systems used in discovery may not be explainable in human terms, all of which complicate regulatory evaluation.

This emerging landscape points to a deeper challenge: the need for regulatory approaches that evaluate not only the final therapeutic product, but also the entire AI-enabled ecosystem that generates, including data pipelines, model validation, algorithmic transparency, and iterative learning loops. To ensure safety, innovation, and equity, future regulatory frameworks must evolve to address the full lifecycle and ecosystem of AI-generated therapeutics, doing so in a coordinated and globally aligned manner. For that, a new framework needs to be defined.

## Toward an integrated regulatory approach

### A framework for AI-Enabled Therapeutics (AI2ET) model

Against a background of regulatory fragmentation and uncertainty, there is a need to reconceptualize how we approach AI oversight for human therapeutics. Regulatory science must keep pace with the integrated nature of AI applications in drug development. It requires that regulation move beyond the conventional paradigm of treating AI as discrete tools by recognizing an interconnected AI ecosystem that covers the entire therapeutic development process.

**Figure 1:**
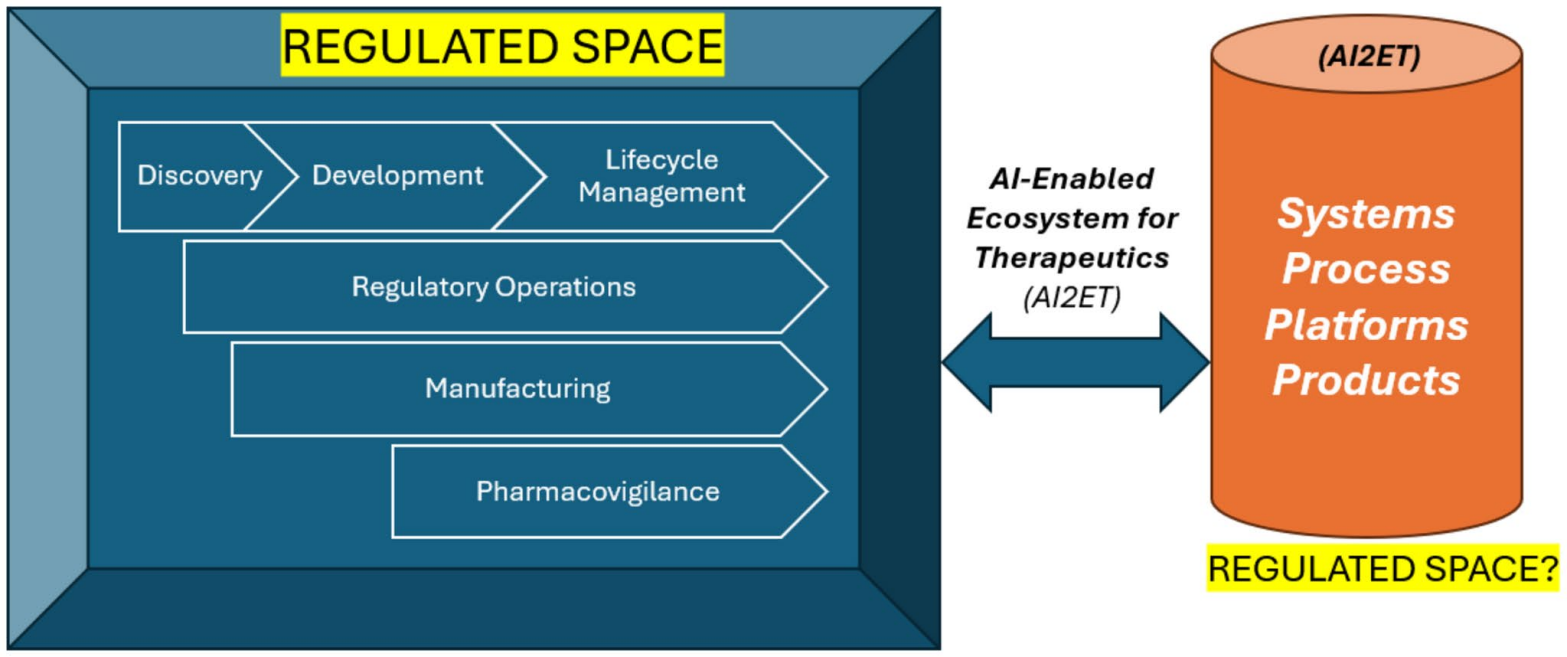
How is *AI-Enabled Ecosystem* for *Therapeutics related* to the various stages of creating human medicines—discovery to lifecycle management (and vice versa).

To address the regulatory fragmentation, we propose the AI-Enabled Ecosystem for Therapeutics, or AI2ET framework. This framework provides a structured process to guide AI oversight across four interconnected components: systems, processes, platforms, and products. Each plays a distinct and interconnected role in enhancing the speed, efficiency, and quality of drug development and regulatory oversight. This framework captures the full range of AI-driven tools, workflows, and outcomes in biotherapeutics. This paper introduces the AI2ET framework and proposes a risk-based regulatory approach in the absence of drug regulations. Figure 1 lays out the components of the framework to show how the AI-enabled biotherapeutic ecosystem AI2ET relates to the various stages of creating human medicines from discovery to lifecycle management.

## Defining the components of the AI-Enabled Ecosystem for Therapeutics (AI2ET)

### Systems

In the context of AI2ET, systems are software or hardware infrastructures that incorporate AI algorithms to execute tasks typically requiring human cognition, such as pattern recognition, decision-making, and adaptive learning. These systems are embedded across clinical, regulatory, and manufacturing operations to support continuous and intelligent workflows.

For example, AI systems are increasingly used in regulatory document review and submission, automating the preparation and validation of Investigational New Drug (IND) and New Drug Application (NDA) filings (18). In pharmacovigilance, AI systems enable real-time adverse event detection by processing large volumes of data from electronic health records, spontaneous reporting systems, and social media (19). During the COVID-19 pandemic, such systems proved essential for managing unprecedented volumes of safety data. Additional applications include risk assessment systems that predict compliance and safety issues, and regulatory intelligence systems that track and interpret evolving global regulatory guidelines. Digital twin systems, virtual models linked to real-time data, are also emerging to simulate and optimize manufacturing processes and therapeutic responses (20). The use of AI and informatics methods to assess how drugs move through regulatory and translational pathways is an example of systems taxonomy (21).

### Processes

Processes refer to the structured series of steps wherein AI is deployed to perform specific tasks in drug development, clinical operations, and quality management. These include routine and complex workflows that benefit from automation, prediction, and continuous improvement enabled by AI.

Prominent examples include AI-driven automation of regulatory submissions, which streamlines the generation and electronic submission of documents while reducing human error. In clinical trials, real-time AI monitoring processes flag protocol deviations and emerging safety concerns, enabling earlier intervention (22). AI also supports intelligent risk management by analyzing operational and historical data to identify high-risk trial sites or patient populations. In pharmacovigilance, AI enhances post-market surveillance by rapidly identifying adverse events and facilitating timely reporting (23). Similarly, in quality assurance, AI processes analyze manufacturing and clinical data to predict quality deviations and improve audit preparedness (24).

### Platforms

Platforms are integrated environments that provide the tools, infrastructure, and services necessary to build, deploy, and manage AI applications at scale. These platforms often support multiple systems and processes across different stages of the therapeutic lifecycle.

In clinical development, platforms can centralize trial and regulatory data, enabling unified oversight and compliance tracking (25). AI-enabled pharmacovigilance platforms monitor real-world data streams, including electronic health records, literature databases, and social media, to automate signal detection (26). Modeling platforms, such as those supporting physiologically based pharmacokinetic (PBPK) simulations, utilize AI to predict drug behavior in varied populations (27). AI-powered document management platforms assist with the automated creation and tracking of regulatory and quality documentation (28). Remote audits can be facilitated by AI or in an augmented reality setting, enabling regulators to access real-time data with minimal need for physical inspections (29). Manufacturing platforms incorporating digital twins facilitate dynamic modeling and control of biologics production, improving process robustness and scalability (30).

### Products

Products are tangible outputs developed, optimized, or manufactured using AI. These include novel drug candidates, vaccines, biologics, and personalized therapies where AI has played a critical role in their design, evaluation, or production.

AI-designed drug molecules, for instance, leverage generative models to create and refine therapeutic candidates in silico before experimental validation (31). During the development of mRNA-based COVID-19 vaccines, AI tools were utilized to optimize mRNA sequence stability and immunogenicity (32). In synthetic biology, AI supports the engineering of microorganisms to produce therapeutic proteins or bio-based materials. Notably, AI-enabled protein design has been recognized with the Nobel Prize, exemplified by the prediction of protein folding and interaction networks for therapeutic applications (17). In advanced therapies, such as autologous cell therapies or gene therapies, AI is increasingly used to support real-time decision-making during manufacturing, enabling personalized control of highly variable and sensitive processes (33).

In the AI2ET framework, the boundaries between systems, processes, platforms, and products are conceptually distinct but often overlap in practice, especially in integrated AI-enabled environments. For example, a single machine-learning model may function simultaneously as a platform, operate within a cloud-based infrastructure (system), drive adaptive trial workflows (process), and influence clinical endpoints that shape the therapeutic product. These overlaps can lead to both synergies and regulatory challenges. While these overlaps are not just technical, they have regulatory implications. For example, the systems frequently house or execute multiple processes, making it challenging to separate infrastructure from function. Platforms that integrate various tools and workflows may embed both systems and processes, actively shaping how tasks are carried out. These tools can also directly influence or even become part of the final therapeutic product, especially in cases like AI-optimized manufacturing or software as a medical device. Validation, change control, and oversight may differ depending on how a tool is categorized. As a result, changes to one component, such as a process within a system, can have downstream effects on the product itself. As AI ecosystems become more integrated, regulators and developers must carefully define these components, not just for clarity, but to align with risk-based frameworks and ensure regulatory application across the full lifecycle.

While distinctions between systems, processes, platforms, and products can quickly blur, the AI2ET framework reframes regulation by treating AI not as a standalone tool but as an ecosystem embedded across the drug development lifecycle. Traditional approaches, such as the FDA’s product-centered paradigm, emphasize the final therapeutic while generally overlooking upstream risks introduced by biased datasets, opaque algorithms, or automated processes. By mapping risks across all layers, AI2ET reduces blind spots that conventional frameworks often miss.

### Risk-based regulatory decision framework

**Figure 2:**
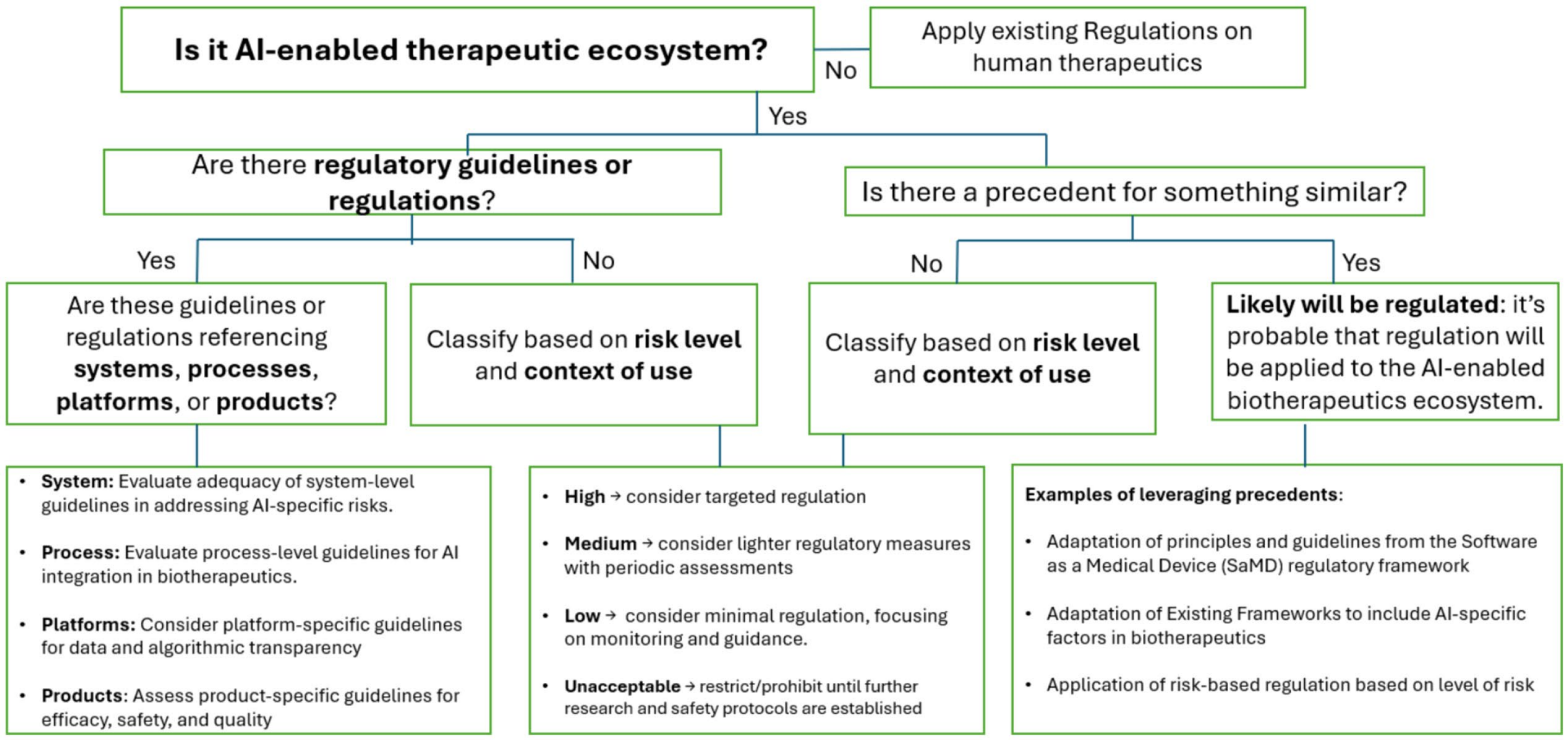
To regulate or not to regulate *AI-Enabled Ecosystem for Therapeutics (AI2ET)*.

AI2ET consists of a risk-based decision framework tailored to guide regulatory decision-making. As shown in Figure 2, this model facilitates context-aware regulatory decision-making aligned with the complexity and potential impact of AI-enabled systems, processes, platforms, and products.

The framework begins by assessing whether existing guidelines apply and, if not, whether regulatory precedents such as FDA’s regulatory guidelines for AI/ML SaMD can be adapted. An example is the FDA’s principles of good machine learning practice (GMLP) applicable to the development of AI/ML-enabled medical devices. One principle is the application of good software engineering and security practices in the development of AI tools. Another principle is the monitoring of deployed models over time to maintain safety and performance. When no precedent exists, AI applications should be classified based on their context of use and potential risk to patient safety or product quality. Risk levels, categorized as high, medium, low, or unacceptable, then inform the appropriate degree of regulatory oversight, ranging from guidance to full premarket review. Figure 2 illustrates the regulatory decision-making process. By grounding regulatory decision-making in the context of use and oversight with potential risk, this framework offers a flexible yet rigorous approach to determining how to regulate, or not, the evolving landscape of AI-Enabled Ecosystem for Therapeutics.

This integrated approach combines elements from existing regulatory guidelines to address AI-enabled drug development. In the absence of specific AI guidelines for therapeutic development, AI2ET bridges between established device AI frameworks and emerging therapeutic AI applications. It addresses the existing regulatory gap where similar AI technologies receive different oversight levels depending on whether they are used in drug development or medical AI applications, even if they face the same technical challenges and risk profiles.

#### How AI2ET Al aligns with some existing ICH guidelines

Regulation under the ICH multidisciplinary guideline M7 on Assessment and Control of DNA Reactive (Mutagenic) Impurities in Pharmaceuticals reflects this recognition (34). Because mutagenic impurities pose direct patient risk, Quantitative Structure–Activity Relationship (QSAR) (35) predictions cannot stand alone. Instead, ICH M7 requires two complementary models, typically one statistical and the other expert rule-based, applied within a defined applicability domain and interpreted with expert judgment. This layered approach ensures transparency, validation, and scientific credibility.

In practice, ICH M7 already mirrors the AI2ET (Artificial Intelligence–Enabled Ecosystem for Human Therapeutics) perspective, treating QSAR not as an isolated tool but as part of a regulatory ecosystem where systems, processes, and platforms converge to safeguard the integrity of the final product.

As an application of AI2ET, QSAR moves from being a black-box model upstream to a regulated ecosystem element with risk-based oversight. This makes the framework actionable where regulators can have a scoring method, oversight pathway, and integration into existing regulations or ICH guidelines.

#### How AI2ET aligns with current regulatory frameworks: US and EU perspectives

As AI is increasingly embedded across the drug development lifecycle, and with evolving regulations, the FDA and the European Union (EU) are adopting risk-based regulatory approaches to govern its use (16, 36). The AI2ET framework, which comprises AI-enabled systems, processes, platforms, and products, aligns naturally with this regulatory shift, as each component introduces different levels of complexity and risk.

In the United States, the FDA applies a tiered, use-case-driven approach. For AI tools used in drug development (e.g., clinical trial design, manufacturing, pharmacovigilance), oversight is generally indirect, relying on existing frameworks such as Good Clinical Practice (GCP), Good Manufacturing Practice (cGMP), and the Drug Development Tool (DDT) Qualification Program (8). When AI outputs directly affect patient care or regulatory decision-making, such as Software as a Medical Device (SaMD) or real-time dosing tools, they are subject to more stringent, direct regulation, requiring defined validation protocols and continuous performance monitoring (7). The FDA’s draft guidance on “Using Artificial Intelligence and Machine Learning in Drug and Biological Product Development” emphasizes context of use (CoU) and risk stratification to determine the appropriate regulatory expectations for AI tools based on their potential impact (6).

In the EU, the regulatory environment is shaped by the EU Artificial Intelligence Act (AI Act), which introduces a comprehensive risk-based classification for AI systems (5). Under the AI Act, AI systems deployed in applications in drug development and therapeutics may be classified as high-risk AI systems, particularly when used for clinical decision-making, safety monitoring, or manufacturing control. These AI systems would then be subject to the requirements of high-risk systems, including transparency, data governance, human oversight, and the implementation of a risk management system. Additionally, existing EMA guidelines, such as those related to the use of modeling and simulation in drug development and quality assurance, provide a foundation for evaluating AI-enabled components of AI2ET.

#### Leveraging the medical devices precedent

Established risk-based principles in medical device regulation provide established precedent on regulatory decision-making within the AI2ET conceptual framework. For example, if a context of use is determined to be low risk from the decision tree (Figure 2), the level of regulatory control may simply be documentation and adherence to general QMS requirements. A medium risk determination would require additional regulatory controls such as performance standards in addition to adhering to general QMS requirements. A high-risk determination would require additional validation through increased amount and rigor of evidence. The 2021 guiding principles of Good Machine Learning Practice (GMLP) for Medical Device Development (37) recommend best practice to address the unique nature of development of AI/ML-enabled medical devices. This framework can be adapted from medical device AI regulation to applications of AI in drug development. The level of risk would determine specific practices from foundational good software engineering practices and data management for all risk types to AI-human interactions in contexts where AI outputs have a significant impact on regulatory decisions.

Together, these frameworks support the application of graduated regulatory oversight based on the role and risk profile of AI tools within the AI2ET ecosystem. For example, an internal AI system used for predictive risk modeling may require only documentation under QMS (38), while a platform used for real-time clinical decision support would need extensive validation and potentially premarket approval (28). This alignment between AI2ET and regulatory risk-based models facilitates innovation while ensuring safety, efficacy, and accountability across AI-enabled therapeutics.

## Policy recommendations: advancing risk-based regulation for AI-Enabled Ecosystems for Therapeutics (AI2ET)

### Clarifying the definition of AI for health regulatory sciences

The FDA has multiple definitions for AI and broadly as “a machine-based system that can, for a given set of human-defined objectives, make predictions, recommendations, or decisions influencing real or virtual environments” (9). While this reflects AI’s interdisciplinary scope encompassing multiple fields such as computer science, statistics, engineering, and decision sciences, such a wide definition introduces ambiguity, scope creep, and difficulty maintaining clear, focused regulatory objectives. We recommend defining AI and machine learning (ML) specifically for health regulatory contexts, with distinct categories for predictive AI and generative AI, and aligning these with the CoU to prevent confusion with competing definitions from other government agencies involved in healthcare AI initiatives (39).

### Modernizing the context of use (CoU) framework

The traditional CoU framework and definition are often too static for the dynamic, adaptive nature of AI tools. AI systems that generate novel outputs or evolve over time pose challenges for a regulatory system that relies heavily on precedent. Without a comparable legacy tool or established regulatory path, the FDA must decide whether to reject an AI-driven tool, accept it based on human interpretation of outputs, or force-fit it into outdated structures. We recommend updating the CoU model to be more modular and adaptable, using the AI2ET framework to capture the complexity and fluidity of modern AI tools.

### Broadening regulatory scope to include discovery and regulatory operations

Current regulatory focus often excludes early-stage drug discovery and operational efficiencies, such as regulatory automation, unless they directly impact patient safety, product quality, or clinical data integrity. However, these AI applications can significantly influence downstream outcomes and regulatory decisions. We recommend that the AI2ET framework explicitly incorporates early discovery tools and AI-driven operational systems that impact therapeutic development quality, reproducibility, or regulatory compliance.

### Leveraging existing insights from medical device AI regulatory developments

There is limited cross-referencing between therapeutic AI regulation and existing AI regulatory framework and insights for SaMD. For example, regulatory insights from the FDA Digital Health Advisory Committee’s November 2024 meeting on generative AI in medical devices offered important insights on premarket evaluation, bias and hallucination risks, model transparency, and post-market monitoring (40). As the regulations for the AI-enabled human therapeutic ecosystem are being developed, incorporating the lessons from the device regulations will ensure consistency across domains and help avoid duplication of effort or regulatory fragmentation.

### Applying risk-based monitoring and scientific judgment

The FDA’s draft guidelines establish a risk-based credibility assessment framework, emphasizing the validation of context-specific models. However, a core question remains: how will AI-generated medicines or outputs be regulated without available guidelines? In such cases, the FDA often relies on Generally Accepted Scientific Knowledge (GASK) (41). Yet, given the “black box” nature of many AI models, GASK alone may not be sufficient; its optimal use will depend on expert human judgment to define what qualifies as “scientific” and what constitutes reliable “knowledge.”

### Use decision tools in the absence of guidelines

To support decision-making where formal regulations are lacking, we propose using a decision tree (see Figure 2). This decision support tool outlines a logical framework for applying CoU and risk-based principles to novel AI-enabled products and tools in biotherapeutics. The decision tree can be a practical guide for evaluating AI tools not covered by existing regulatory precedents. Here, an expert can serve as the human-in-the-loop in regulatory decision-making by actively guiding the system by applying domain knowledge to support accurate and accountable outcomes.

Implementation of these recommendations could proceed in phases, such as piloting use cases under existing guidelines, harmonizing AI/ML terminology relevant to human therapeutics by seeking consensus at the ICH level, or establishing an advisory board modeled on those used for medical devices and generative AI (40). Alternatively, a more ambitious approach would be to create a new, agile regulatory authority dedicated to overseeing the AI-enabled ecosystem for human therapeutics (42).

## Limitations of AI2ET

It is recognized that the AI2ET framework, as a novel construct, will often operate outside the traditional applicability domain (AD) established for computational models. Regulatory precedents, such as those governing Quantitative Structure–Activity Relationship (QSAR) models, already mandate the submission of a clearly defined AD. Predictions made outside of this domain are considered to carry higher uncertainty and typically require additional biological or non-clinical studies for validation. We therefore acknowledge this precedent: out-of-domain predictions should not be dismissed but rather escalated for enhanced oversight, validation, and confirmatory evidence. This principle offers a practical and well-established pathway that can be extended to AI2ET, ensuring proportionate regulation when models are applied beyond their validated boundaries. More research needs to be conducted on this topic.

AI2ET framework also faces several limitations in offering a promising model for federating and harmonizing AI-related drug regulatory knowledge. First, integration into the existing global regulatory environment remains challenging due to wide disparities in digital infrastructure, data governance norms, and regulatory maturity across countries. Many low- and middle-income regions may lack the resources, technical expertise, or institutional capacity to effectively adopt and apply AI2ET-derived insights, potentially reinforcing existing inequities. Additionally, real-time data ingestion and multilingual processing require sustained computational resources and maintenance, which may limit scalability. There is also a risk that over-reliance on automated systems could lead to regulatory inconsistencies, particularly in areas where qualitative judgment, cultural context, or legal nuance is essential. Finally, without formal recognition or endorsement by major regulatory bodies, the framework may struggle to gain traction or influence decision-making on a scale. For AI2ET to reach its full potential, strategic partnerships, transparency, and continuous validation will be essential to ensure its reliability, adaptability, and global relevance.

## Conclusion

As artificial intelligence becomes increasingly central to drug development, regulatory frameworks must evolve to keep pace. The AI-Enabled Ecosystem for Therapeutics (AI2ET) provides a structured, drug lifecycle-wide approach to managing AI’s role in discovery, clinical development, manufacturing, regulatory enabling functions, and post-market oversight.

However, current regulatory guidance often falls short, limited by definitions of AI and CoU’s, narrow scope, and lack of integration with digital health and device regulations. To close these gaps, a risk-based, context-aware model grounded in AI2ET is needed. This includes redefining AI for regulatory purposes, updating CoUs for adaptive tools, and broadening the scope of oversight across domain regulatory efforts. The proposed decision tree is a practical tool to guide regulatory decisions, especially when formal precedents are absent.

The AI2ET framework is not intended as an abstract construct but as a policy tool anchored in existing regulatory practice. For example, ICH M7’s structured use of QSAR models already embodies the AI2ET principle by regulating the broader ecosystem—the systems, processes, platforms, and products, rather than only the final outcome. AI2ET builds on such precedents by offering a risk-based decision tree that regulators and industry can operationalize today. Far from remaining theoretical, it provides a pathway for a harmonized, adaptive regulatory oversight that integrates upstream AI tools into the existing review structure.

Under AI2ET, AI is defined not as a standalone tool, but as an embedded, evolving capability that influences safety, efficacy, quality, and compliance across the therapeutic ecosystem. This approach ensures oversight focuses on function and impact, rather than the technology itself, supporting innovation while safeguarding public health in an AI-driven era. In this emerging paradigm, AI is no longer viewed as a discrete tool, but as an embedded, evolving capability interwoven into platforms, systems, and decision-making processes that span the entire drug lifecycle.
